# Gender differences in the health status of elderly living alone compared to those who are not alone: Evidence of the AHAP study, North of Iran.

**Published:** 2016

**Authors:** Simin Mouodi, Ali Bijani, Seyed Reza Hosseini, Karimollah Hajian-Tilaki

**Affiliations:** 1 Social Determinants of Health Research Center, Health Research Institute, Babol University of Medical Sciences, Babol, Iran.; 2 Department of Biostatistics and Epidemiology, Babol University of Medical Sciences, Babol, Iran.

**Keywords:** Living alone, Elderly, Health status, Iran.

## Abstract

**Background:**

One of the factors that have impact on the health status of elderly people is living alone. This study was conducted to examine the living condition of elderly population in Babol and probable differences which this condition induced on the health disorders of elderly people.

**Methods:**

This cross-sectional study was performed on 1544 elderly people aged 60 and over in Amirkola, Babol. Demographic characteristics, the incidence of falls, chronic diseases, polypharmacy, headache with uncertain cause, chronic pains and back pain were collected by the study questionnaire; social support, physical activity, depression and cognitive disorders were assessed with standard questionnaires.

**Results:**

6.8% of elderly people were living alone. Lack of social support, cognitive disorders, depression, multiple chronic diseases, occurrence of falls and headache with uncertain cause were significantly higher among those elderly who live alone (p<0.05). Regardless of age and educational level, headache and depressive symptoms in male individuals living alone, and falls occurrence in female individuals were significantly more than those who did not live alone (p<0.05). Also, in female elderly subjects, the effect of the marital status factor on health-related disorders was more than the effect of living alone factor.

**Conclusion:**

Many disorders and disabilities are higher in the elderly people who live alone; also there is a difference in the health status of elderly people who live alone, according to their gender.

**A**ccording to the publications of WHO, people aged 60 years and older are characterized as old people (1) and accordingly, the elderly are divided into three groups: “young old” (60-74), “old old” (75-84) and “oldest old” (85 years and older) (2). Since the elderly population is growing in the world and in Iran, health care providers should get familiar with the different aspects of health-related issues of aging population as an important indicator of their activity and to design proper interventions to identify the risk factors and prioritize the significant health needs (3, 4). One of the factors that can affect the health status of elderly and put them in an “at- risk” group to different disorders and disabilities is living alone (5, 6). In Western societies, the number of elderly who are living alone has increased, but despite this upward trend, there is limited information available about the overall experience of living conditions of this population (7).

Living alone can affect the mental health and quality of life (8), causing or worsening symptoms of depression (9, 10). On the first phase of AHAP study (11), among 1616 elderly people of Amirkola, 1379 (85.3%) individuals were married and 14.7% were living without a partner. In this study and subsequent researches, the health status of the elderly people was examined in various aspects, including chronic diseases (12), depression (13), osteoporosis (14), cognitive disorders (15, 16), medications (17), the relationship between social support and depression (18), the association between musculoskeletal pain and disability (19), the link between chronic disease and disability in the elderly (20), and others; but their living arrangements and the impact of living alone on health, comorbidity and disability have not been evaluated. Therefore, in this study, the living arrangements of the elderly in Amirkola and possible differences of situations associated with living alone, between males and females, its impact on health problems (including physical disorders, incidence of falls, depression, cognitive impairment, chronic pain and polypharmacy) were examined.

## Methods

This observational analytical research was carried out with a cross-sectional study on 1544 elderly aged 60 years and older participating in the first phase of AHAP study in North of Iran (11). Using the address of this elderly population available in two health centers in Amirkola, all the individuals were contacted via phone or home visit. In addition to giving information about the different aspects of the study including the aims and methods, the elderly people were invited to participate in the research plan and refer to the Population Research Center of Amirkola. Study samples were enrolled from the target population using convenience sampling. Demographic characteristics (sex, age, educational level and marital condition), occurrence of falls in the past 6 months, chronic disease (according to the patients’ medical records and drug prescriptions), polypharmacy (using 4 drugs or more), unexplained headaches, chronic pain lasting more than three months in the last 6 months and the thoracic and lumbar pain during the past 12 months were collected using questionnaires. Social support was assessed with Duke Social Support Index (DSSI); physical activity was evaluated using Physical Activity Scale for the Elderly (PASE); depression with Geriatric Depression Scale (GDS) and cognitive impairment was assessed with Mini-Mental State Examination (MMSE). Duke Social Support Index (DSSI) contains 11 questions in the areas of social interaction (4 questions, scores 4-12) and the satisfaction of social interaction (7 questions, scores 7-21) to measure various aspects of social support. Higher scores indicate greater social support. Validity and reliability of the questionnaire have been approved in the general population and the elderly population inside the country and other studies (18, 21, 22). 

Physical Activity Scale for Elderly (PASE) contains 12 questions which describe a variety of physical activities preferably selected by elderly, such as walking, recreational activities and sports, home and yard activities and taking care of others. The frequency, duration and intensity of activity over the past week were evaluated and points ranged 0-400 were obtained. Higher score indicates more physical activity. This questionnaire was used in previous studies (23). Geriatric Depression Scale (GDS) contains 15 questions, in which score of 6 or above indicates depression in the elderly. Its validity and reliability for screening the depression symptoms in the elderly have been approved (13, 24, 25). Mini-Mental State Examination (MMSE) was used to assess the cognitive performance of the subjects. The questionnaire evaluates various cognitive aspects, including attention, orientation, memory, calculation and language. Scores less than 25 out of a total of 30 points considered as cognitive impairment. Validity and reliability of the questionnaire have been approved inside and outside the country (15, 16, 26, 27).

Also, unknown hypertension in the elderly with no history of medical diagnosis was defined after a physical examination and systolic blood pressure greater than or equal to 140 or diastolic blood pressure greater than or equal to 90 in two blood pressure measurements; unknown diabetes with no history of medical diagnosis of diabetes was described after two venous blood sampling and fasting blood glucose ≥ 126 (28). Participation in the study was voluntary and the elderly could refuse whenever she/he wanted to. In this case, refusing to or participate or withdrawing from the study any time did not affect their rights and health. In addition, all necessary precautions were carried out to respect the privacy and confidentiality of information about samples.

The collected data were analyzed by SPSS Version 16. In bivariate analysis of data, chi-square test was used, in addition, multiple logistic regression model was applied to assess the impact of living alone on health condition. In this model, main independent variable was living alone which is defined as indicator variable, and the dependent variable was associated comorbidities and disabilities, depression and cognitive impairment which were separately entered into the model as dependent variables. The adjusted odds ratio of living alone (versus not living alone) was estimated by controlling age and educational level and its 95% CI was calculated. In addition, logistic regression was performed separately for males and females to assess the role of gender as a moderating variable to what extent can modify the association of living alone on health conditions. Meanwhile, in addition to living alone, to examine the impact of marital status on the outcomes of old age, marital status, as an indicator was also entered into the model. 

## Results

One hundred five cases (29 men and 76 women) out of a total of 1,544 elderly were living alone. The percentage of elderly participant living alone was 6.8% (95% CI: 5.54- 8.06%) which was 3.45% in men (2.21- 4.68%) and 10.81% in women (8.5-13.10%), respectively (P<0.001). The demographic information, the status of comorbid diseases and disabilities among elderly who live alone versus to those who do not live alone, are given in table 1. Among the 231 subjects (60 males and 171 females) who were unmarried, 91 (39.4%) cases were living alone and 14 cases, despite being married, were also living alone. The impact of living alone on comorbid diseases and disabilities was different in the two sexes. Data analysis showed that living alone had a significant impact on cognitive impairment and depression in men; and on the occurrence of falls in women. The adjusted odds ratio (OR) for age and educational level were calculated using logistic regression model to evaluate the association between living alone and comorbid diseases and disabilities separately for two sexes, which is presented in table 2. Since marital status affects the establishment of comorbid diseases and disabilities, the logistic regression model was performed using the Backward method by adding marital status to the previous variables, wherein no significant differences were observed in men, but in women the importance of marital status was more highlighted compared to living alone; consequently, living alone had a significant effect on falls occurrence in females, but being single had a significant association with the number of chronic diseases (OR = 1.507,95%CI:1.026-2.214, P=0.036), depression (OR=1.865, 1.281-2.717, P=0.001) and chronic pain for more than three months during the last six months (OR=4.451, 1.418 -13.970, P=0.014).

**Table 1:** Demographic information and comorbidities among elderly who live alone compared with other elderly

**p-value**	**Elderly who live alone** **N=105**	**Elderly who don’t live alone** **N=1439**	**Demographic variables and comorbid diseases and disabilities**
0.000	29 (27.6) 76 (72.4)	812 (54.6) 627 (43.6)	Gender: male (%) Female (%)
0.000	73.62±7.25	60.05±7.33	Age (mean±SD)
			Educational Status:
0.206	76 (72.4) 25 (23.8) 4 (3.8)	927 (64.4) 413 (28.7) 99 (6.9)	Illiterate (%) Primary and secondary education (%) High school and higher education (%)
0.069	32 (30.5)	327 (22.7)	Polypharmacy
0.071	96 (91.4)	1223 (85)	Chronic pain in the past 6 months (%)
0.431	70 (66.7)	904 (62.8)	Back pain in the past 12 months (%)
0.004	57 (54.3)	574 (39.9)	Unexplained headaches (%)
0.792	28 (26.7)	367 (25.5)	Undiagnosed diabetes or hypertension (%)
0.009	64 (61)	687 (47.7)	3 or more chronic diseases (%)
0.006	29 (27.6)	245 (17)	The falls occurrence in the past six months (%)
0.000	65 (61.9)	606 (42.1)	Symptoms of depression (%)
0.000	6.49±3.96	4.45±3.40	GDS score (mean±SD)
0.002	48 (45.7)	444 (30.9)	Cognitive impairment (%)
0.005	24.11±3.75	25.24±3.95	MMSE score (mean±SD)
0.233	98.65±51.91	106.10±62.37	PASE score (mean±SD)
			DSSI score (mean±SD)
0.000 0.004 0.000	25.61±3.96 9.79±1.62 15.82±3.49	27.61±3.20 10.26±1.41 17.34±2.64	Social interaction score (mean±SD) Score of Satisfaction of social interactions (mean±SD)

Table 2 shows that living alone had a statistically significant correlation with unexplained headache, multiple chronic diseases, the occurrence of falls and depression symptoms. Living alone in men is associated with unexplained headache and depression symptoms, while in women, only with the occurrence of falls.

**Table 2:** The adjusted odds ratio (OR) for age and education level and comorbidities divided for two sexes

**Female**		**Male**		**Total**		**Comorbid diseases and disabilities**
**pvalue**	**OR (95% confidence interval** **)**	**pvalue**	**OR (95% confidence interval** **)**	**pvalue**	**OR (95% confidence interval** **)**	
0.709	1.10 (0.66-1.84)	0.82	1.12 (0.41-3.05)	0.088	1.47 (0.95-2.28)	Polypharmacy
0.885	0.94 (0.37-2.34)	0.304	1.9 (0.56-6.42)	0.116	1.76 (0.87-3.57)	Chronic pain in the past 6 months
0.572	0.85 (0.48-1.50)	0.316	0.68 (0.32-1.45)	0.493	1.16 (0.76-1.78)	Back pain in the past 12 months
0.392	1.25 (0.75-2.07)	0.040	2.25 (1.04-4.87)	0.001	2.03 (1.35-3.05)	Unexplained headaches
0.929	0.97 (0.53-1.77)	0.216	1.62 (0.75-3.49)	0.960	0.99 (0.63-1.56)	Undiagnosed diabetes or hypertension
0.801	1.07 (1.03-2.96)	0.141	1.76 (0.83-3.75)	0.007	1.76 (1.17-2.65)	3 or more chronic diseases
0.040	1.74 (1.03-2.96)	0.514	0.7 (0.23-2.07)	0.029	1.67 (1.05-2.63)	The falls occurrence in the past six months
0.265	1.35 (0.8-2.28)	0.022	2.43 (1.14-5.18)	0.000	2.2 (1.46-3.35)	Symptoms of depression
0.704	0.90 (0.53-1.55)	0.630	1.34 (0.53-2.83)	0.115	1.42 (0.92-2.21)	Cognitive impairment

## Discussion

The results showed that lack of social support, cognitive disorders, depression, multiple chronic diseases, occurrence of falls and unexplained headache in elderly people who live alone are significantly more, also, regardless of age and level of education in male individuals who live alone, unexplained headache and symptoms of depression and in females who live alone, occurrence of falls are significantly higher than those not living alone. On the other hand, in females, the role of marital status was more highlighted than the living alone variable, thus, living alone was only effective in occurrence of falls, however, being unmarried had impact on the occurrence of multiple chronic diseases, depression and chronic pain. These results showed the differences between the two sexes in the incidence of diseases and disabilities in elderly people who live alone.

The mean age of the elderly who lived alone was higher, and in terms of gender of lonely elderly, females were more; these findings are similar to the study of Kharicha in UK (6). In Kharicha’s study, the elderly who lived alone reported poorer health situation, weaker eyesight, daily life problems, impaired memory, bad temper, lower physical activity, poor diet, weaker performance, more social isolation, increased use of alcohol, lack of emergency care and high incidence of falls during the past 12 months. The result of more depression and lack of social support in elderly who live alone is consistent with the study of Tang in China (9), which stated that more than 30% of people aged 60 years and over in Shanghai who lived alone reported depressive symptoms and after controlling the demographic variables and health status, social isolation was significantly associated with depression symptoms. 

In the study of Dean at the University of San Diego (10) on a population aged 50 years and over, the results showed that elderly people who lived alone had a higher level of depressive symptoms, and the association between the two variables of loneliness and depression were independent of other factors, including the support of friends, face to face communication with friends, life events, disability and financial distress. In the study of Dean, the impact of depression was more in men than women, which is consistent with the findings of the present study. Additionally, unwanted health complications had a greater impact on those who live alone, especially on women, while in our study only the incidence of fall was significantly higher in elderly women who lived alone. In the study of Henning-Smith in USA (8) on community- dwelling subjects aged 65 and over (out of elderly care institutions), the results showed that although the quality of life was poorer and psychological distress was more in elderly people who lived alone than those who lived with someone else, but the effects of these conditions were different in two sexes: women who lived with others (other than spouse) were more seriously at higher risk of poor quality of life and psychological distress than men, and vice versa in the study of Greenfield, USA (5), living alone was associated with feeling more loneliness in men than women. While in our study, men who lived alone had worse situation than women regarding depression and psychosomatic symptoms such as unexplained headaches; on the other hand, the elderly women who did not have husbands had more chronic diseases, depression and chronic pains than older married ones. 

In this study, social support (in both areas of social interaction and satisfaction of this interaction) in elderly people who were living alone was significantly more inappropriate than those who were not living alone, but hypertension was not statistically different between the two groups; this finding has differences with the results of the study of Yang in USA (29) which stated that low social support is a predictor for systolic hypertension in the elderly and socially isolated elderly people are at increased risk of hypertension.

In this study, the observed differences between the sexes in terms of the effect of living alone on comorbid diseases and disabilities could be due to differences between the two sexes in terms of social and interpersonal relationships and social situations. The study of Hemati in Tehran (30) pointed out that in the elderly, inability to maintain normal lifestyle leads to feel loneliness, isolation and subsequent physical and psychological effects. In traditional Iranian culture, men generally have more social activities and broader communication than women, and as the head of a family, they make more important decisions. The quality of these relations is reduced by living alone and on the other hand, many previous occasions, such as management and decision-making authority are lost and these changes can influence on the subsequent physical and mental disorders. Besides, feeling loneliness in the elderly does not depend on the frequency of relationships with children and friends, but the quality of these relationships, expectations and satisfaction of these communications are important; elderly people who do not meet the expectations of visiting family members and friends and are not satisfied when communicate with them, feel loneliness and the complications of this loneliness are manifested in different aspects of their physical and mental health.

The limitations of this study include lack of assessment of quality of life and feeling loneliness in the elderly and the possible differences between the two groups of being alone and not being alone elderly population in quality of life and feeling of loneliness. Since there were many questionnaires related to the research plan and duration needed for the response, physical examination and testing of blood samples, using other questionnaires to assess the other aspects of health and life quality were impossible for researchers and boring for the participants.

**Conclusion**

Many diseases and disabilities are more in elderly who are living alone compared with those who are not living alone; additionally, the health status of elderly people who live alone is different based on gender. Therefore, it is necessary to promote interventional programs to enhance proper living conditions, social and family interactions of the elderly; identify elderly population who live alone and perform regular screening of physical and mental disorders to prevent the incidence of severe complications of these diseases and increase longevity and quality of life of elderly people.

## Notes

**Funding:** The Vice-Chancellery for Research and Technology of Babol University of Medical Sciences funded this project.
